# Development of a standardized in vitro model to reproduce hydrophilic acrylic intraocular lens calcification

**DOI:** 10.1038/s41598-022-11486-0

**Published:** 2022-05-10

**Authors:** Leoni Britz, Sonja Katrin Schickhardt, Timur Mert Yildirim, Gerd Uwe Auffarth, Ingo Lieberwirth, Ramin Khoramnia

**Affiliations:** 1 The David J. Apple International Laboratory for Ocular Pathology, Department of Ophthalmology, University Eye Clinic Heidelberg, University of Heidelberg, INF 400, 69120 Heidelberg, Germany; 2 Department of Physical Chemistry of Polymers, Max Planck Institute for Polymer Research, Mainz, Germany

**Keywords:** Lens diseases, Eye diseases

## Abstract

Opacification through calcification of hydrophilic acrylic intraocular lenses (IOL) is a severe complication after cataract surgery. Causing symptoms that range from glare through to severe vision loss, the only effective therapy is explantation of the opacified IOL so far. Although IOL calcification is a well-described phenomenon, its pathogenesis is not fully understood yet. The purpose of the current study was to develop a laboratory model to replicate IOL calcification. Calcification could be reproduced using a horizontal electrophoresis and aqueous solutions of calcium chloride and disodium hydrogen phosphate. The analysis of the in vitro calcified IOLs was performed using light microscopy, Alizarin Red and Von Kossa staining, scanning electron microscopy, energy dispersive x-ray spectroscopy and electron crystallography using transmission electron microscopy and electron diffraction. The presented laboratory model could be used to identify hydrophilic IOLs that are at risk to develop calcification and to assess the influence of associated risk factors. In addition, it can serve as a research tool to further understand this pathology.

## Introduction

Cataract surgery with intraocular lens (IOL) implantation is one of the most common performed surgeries worldwide and generally a very safe procedure. Nevertheless, complications exist, which may lead up to a removal of the IOL^1^. A recent publication investigating IOL related complications found opacification of hydrophilic acrylic IOLs through calcification^2–7^ to be the main reason for IOL explantation^8^. Laboratory investigations identified calcification to be the precipitation of calcium and phosphate ions to form hydroxyapatite crystals within the IOL polymer^9–11^. Growth and accumulation of those crystals increases straylight causing symptoms that range from glare through to severe vision loss^12–16^ and IOL explantation and exchange is the only effective therapy so far.

IOL calcification is associated with a number of risk factors: some hydrophilic IOL polymers appear more prone than others to calcification and opacification of IOLs of certain batches could be observed in the past^16^. Moreover, there is an association with some ophthalmic surgical procedures or with specific ocular and systemic diseases^8,9,15–20^.

Although IOL calcification is a well-described phenomenon^2–6^, its pathomechanism and its relation to risk factors is not fully understood yet. The use of in vitro methods is of crucial importance in aiming to understand the underlying mechanism of calcification in order to avoid such pathology. The objective of this study was to develop a standardized in vitro model to reproduce IOL calcification, which is the formation of hydroxyapatite crystals within the IOL polymer. We aimed to create a fast-screening method to identify IOLs that are at risk develop calcification, which further allows to investigate the influence of risk factors of calcification.

## Methods

**Figure 1:**
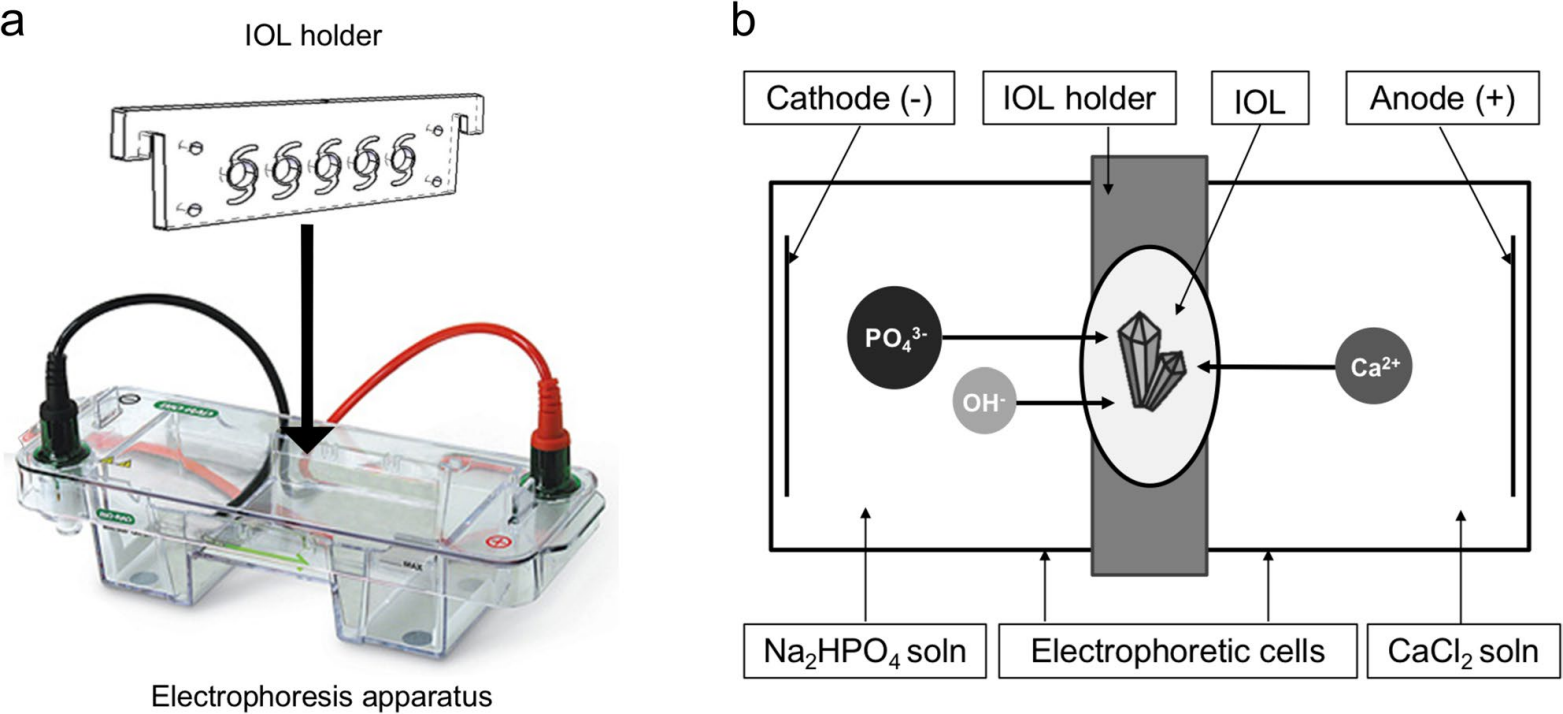
Schematic illustration of experimental setup (**a**) and hydroxyapatite formation within the intraocular lens (IOL) polymer (**b**). (**a**) The IOLs were placed in the double-walled holder having a circular opening exposing the IOL’s surfaces. The holder was then placed in the electrophoresis tank with a rubber seal to avoid leakage of the solutions. (**b**) The disodium phosphate aqueous solution was set at the cathode side and the calcium chloride aqueous solution at the anode side. Calcium cations and phosphate anions migrate toward the corresponding electrode side—passing through the IOL polymer. Hydroxyapatite was formed as the opposing ions met in the gel.

In our experimental model to replicate IOL calcification in vitro, we adapted existing electrophoretic approaches to form hydroxyapatite in hydrogels from Watanabe and Akashi^21^ and Schiraldi et al.^22^ to IOL polymers. We used a Mini-Sub Cell GT Cell horizontal electrophoresis tank with platinum electrodes (Bio-Rad Laboratories Inc., Hercules, California, USA) and a double-walled IOL holder of our design, made out of inert Poly(methyl methacrylate) (Fig. 1a). For each electrophoretic run, five acrylic IOLs from different manufacturers (Table 1) were unpacked and placed in the IOL-holder, taking care to avoid IOL dehydration while transferring them from the pack. The holder containing the IOLs was placed in the electrophoresis tank. Rubber seal was set between the holder and the tank to avoid an exchange of the different salt solutions.

**Table 1:** Intraocular lens characteristics.

P^a^	Manufacturer	Model	Material^b^	H_2_O %
a	Oculentis Medical GmbH	LentisMplus LS-313 MF30	PHEMA	25
b	Rayner Intraocular Lenses Ltd	Centerflex Toric 571 T PCL	PHEMA	26
c	Morcher GmbH	95S	PHEMA	28
d	Carl Zeiss Meditec AG	CT SPHERIS 204	PHEMA	25
e	Alcon Laboratories, Inc	Clareon SY60WF^c^	PEA	1.5

For the IOL models LentisMplus LS-313 MF30 and CT SPHERIS 204, the manufacturers claim these IOLs to have hydrophobic surface properties. There is no precise information given on what these surface properties consist of.

P = position. PHEMA = Poly (2-hydroxyethyl methacrylate). PEA = Poly (2-phenylethylacrylate).

^a^Position in IOL holder (Fig. 1), identical for each IOL model per run.

^b^Major material component specified.

^c^Hydrophobic negative control.

To simulate the aqueous humor in respect of the components forming the calcium phosphate crystals in vivo, aqueous solutions of calcium chloride and disodium hydrogen phosphate of physiological aqueous humor concentration (CaCl_2_ = 1,70 mM, Na_2_HPO_4_ = 0,62 mM), 10 mM and 40 mM concentration were prepared. To adjust the solutions to pH 7,40 we used 10 mM TRIS-buffer and 10 mM HCl-buffer. The calcium chloride aqueous solution was set at the anode side and the disodium hydrogen phosphate aqueous solutions at the cathode side (Fig. 1b). To adapt the electrophoretic approaches to the IOL polymer, five runs with five IOLs each (n = 25) were made and tested with alterations in electrophoresis time, the concentration of the solutions and the applied voltage, until we found the optimal conditions for sufficient crystal growth to perform electron crystallography (Table 2).

**Table 2:** Adaption of electrophoretic settings to the IOL polymer until sufficient crystal size for analysis was produced.

Parameter	Run 1	Run 2	Run 3	Run 4	Run 5
*c,* mM	Na_2_HPO_4_ 0,62 CaCl_2_ 1,70	40^a^	**10**^**a**^	10^a^	10^a^
*t,* h	1	1	**20**^**b**^	4	20^b^
*I,* mA	50	50	**25**	25	25
*U,* V	100	100	**100**	300	100

We started with settings as in Schiraldi et al^22^ and physiological concentrations, then successively adapted the settings to the IOL polymer. In order to enable analysis with electron crystallography, a certain crystal size is required, which was best created by parameter settings in run 3 (bold). These settings were set as standard and verified in run 5. Constant parameters in every run were pH 7,40, power 41 W and temperature 22 °C.

^a^Concentration of both CaCl_2_ and Na_2_HPO_4_ aqueous solutions.

^b^To ensure sufficient ion concentration, the aqueous solutions were changed after 8 h.

**Figure 2:**
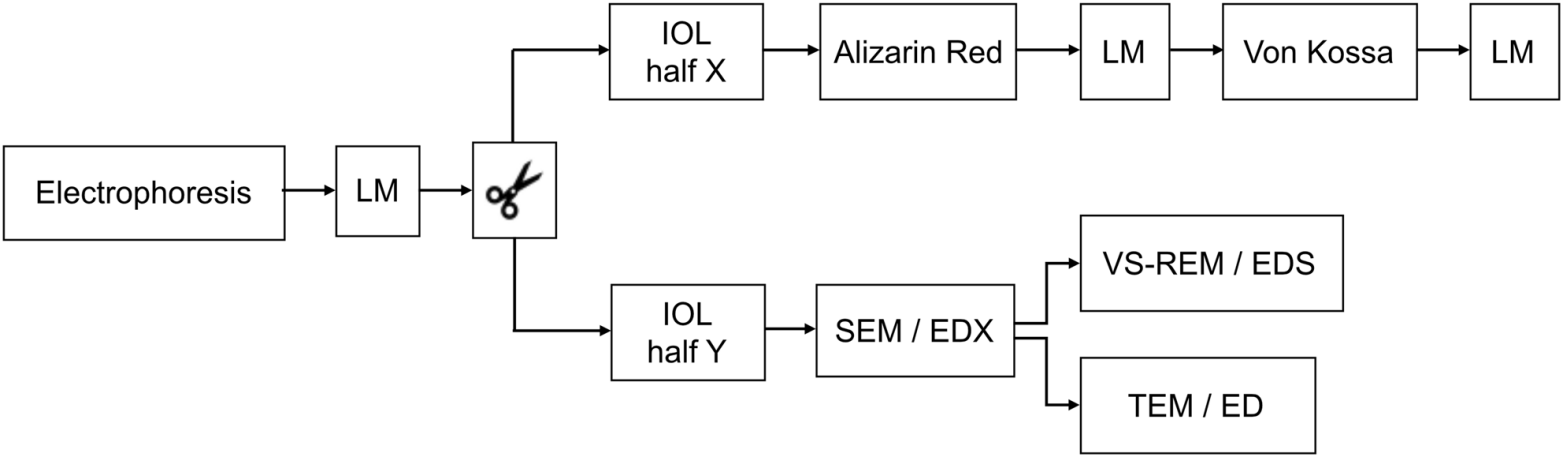
Analysis methods. After electrophoresis, light microscopy (LM) images were taken of the complete IOLs. Then the IOLs were bisected into X and Y halves. The X half was analyzed using the Alizarin Red and the Von Kossa methods. The anterior and posterior surfaces of the Y half and vertical sections (VS) of these Y halves were investigated using scanning electron microscopy (SEM), energy dispersive X-ray spectroscopy (EDX), transmission electron microscopy (TEM) and electron diffraction (ED).

After electrophoresis, the IOLs were removed from the holder, rinsed with purified water and then investigated for the occurrence of calcification. We applied the analysis methods we use to analyze explanted IOLs that we have reported in detail in previous publications^4,9,12–14,17,20^ (Fig. 2): beginning with light microscopy, taking overview images and photographs at higher magnification. Then each IOL was bisected into X and Y halves. The X half was first analyzed using Alizarin Red staining, the histological stain used to detect calcium deposits on the IOL surface. To identify if calcium phosphate deposits had formed within the IOL polymer, this X half was paraffin-embedded, then cut in 5 µm vertical sections and stained with the Von Kossa method, which stains (calcium) phosphates brown to black. For electron microscopy investigations, the Y halves were sent to the Max-Planck-Institute for Polymer Research. Scanning electron microscopy (SEM) images of the anterior and posterior surfaces and vertical sections were taken and chemical analysis of the deposits formed on the surface and within the polymer was performed using energy dispersive X-ray spectroscopy (EDX). Electron crystallography of the deposits within the polymer was carried out, using transmission electron microscopy (TEM) and electron diffraction (ED).

### Ethical approval

The study did not require institutional review board approval because it was an exclusively experimental in vitro study. Besides the authors, no other person contributed to the conduct of this study and the preparation of the manuscript.

## Results

### Adjustment of electrophoretic parameters

Under physiological concentrated solutions and a one-hour running time in run 1 (Table 2), light microscopy and staining methods showed crystals had formed on the IOLs surfaces only, but not within the polymer, resulting in negative Von Kossa analysis. Therefore, concentration of the aqueous solution was increased to 40 mM in run 2, as used by Schiraldi et al.^22^. Under these conditions, Von Kossa staining and SEM with EDX showed crystals had formed within the polymer but were too small to perform electron crystallography. Running time was therefore increased from one hour to 20 h in run 3. Furthermore, the concentration of the solutions was reduced from 40 to 10 mM, to minimize precipitation outside the polymer. Under these settings, a complete crystal analysis including electron crystallography was possible, so we decided to use the settings in run 3 as standard settings in the future. In addition, we tested the influence of higher voltage on crystal formation in run 4. Increasing voltage from 100 to 300 V resulted in inhomogeneous crystal growth restricted to certain areas in the polymer. Optimal crystal formation occurred under the settings used in run 3. The settings and obtained results were repeated and therefore verified in run 5.

### Crystal analysis

**Figure 3:**
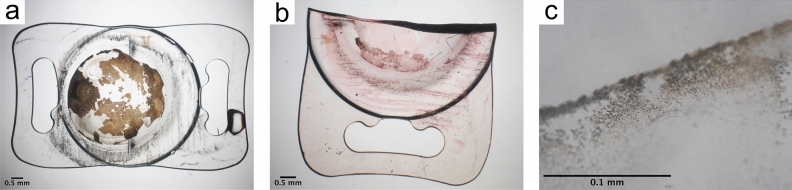
Light microscopy investigation of IOL 3a. Light microscopy (**a**) shows flat and granular deposits on the IOL’s surface. Alizarin Red (**b**) and Von Kossa (**c**) staining reveal the deposits had formed on the surface (**b**) and within the IOL polymer (**c**) and consist of calcium phosphate.

**Figure 4:**
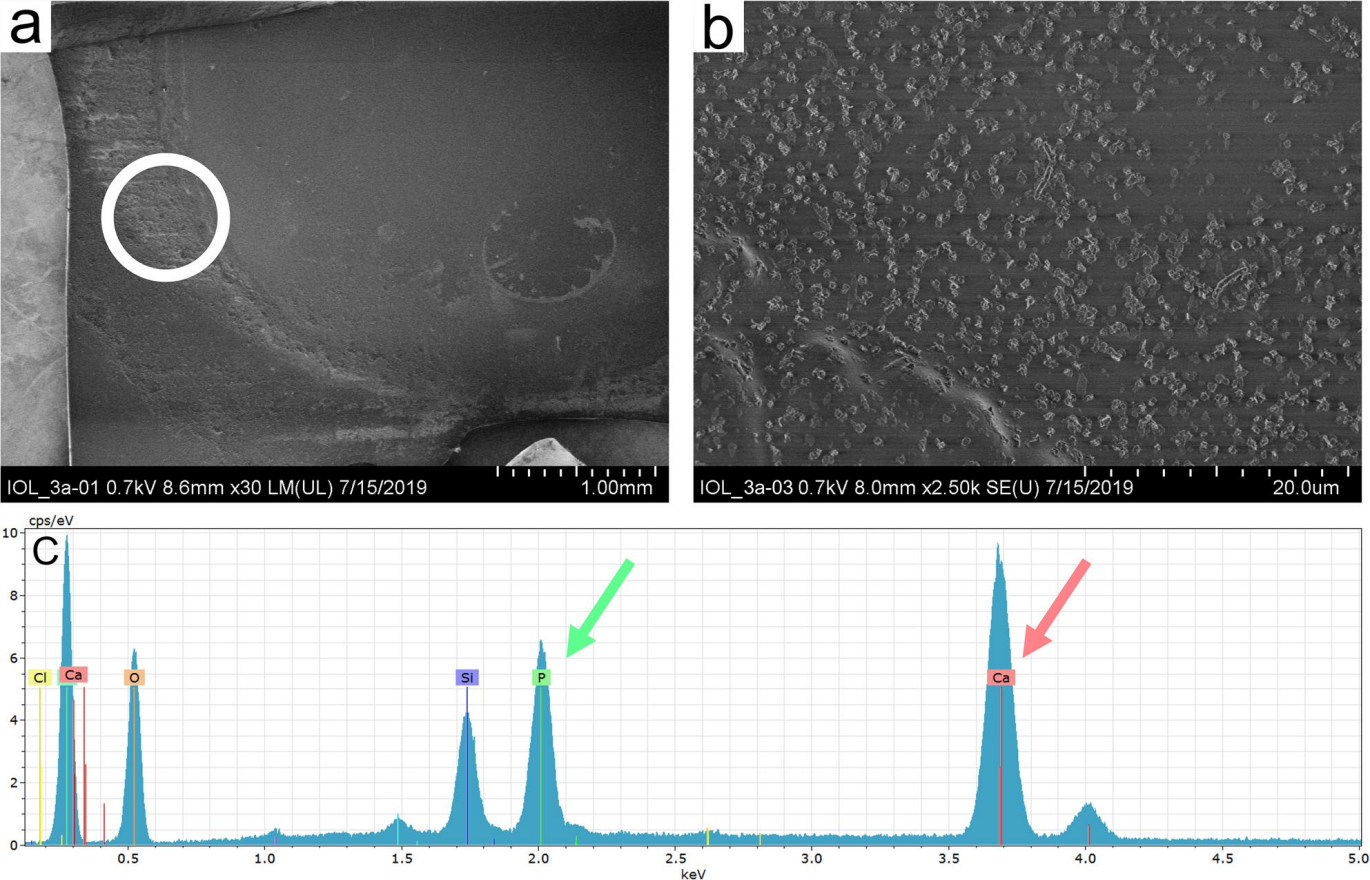
Analysis of surface deposits of IOL 3a. Scanning electron microscopy (**a**, **b**) shows deposits formed on the IOL’s surface. Energy dispersive X-ray spectroscopy (**c**) investigation shows high peaks of 6,5 cps/eV at 2 keV characteristic for phosphorus (P) and 9,5 cps/eV at 3,7 keV characteristic for calcium (Ca). These findings confirm the deposits to be calcium phosphates. Since the sample was placed on a silicon waver for analysis, a silicon peak (Si) can be found in the EDX result. Carbon (C) and oxygen (O) peaks originate from the IOL polymer.

Light microscopy and Alizarin Red staining showed calcium deposits had formed on the IOL’s surface (Fig. 3a,b). SEM with EDX confirmed these deposits to consist of calcium and phosphorus (Fig. 4).

**Figure 5:**
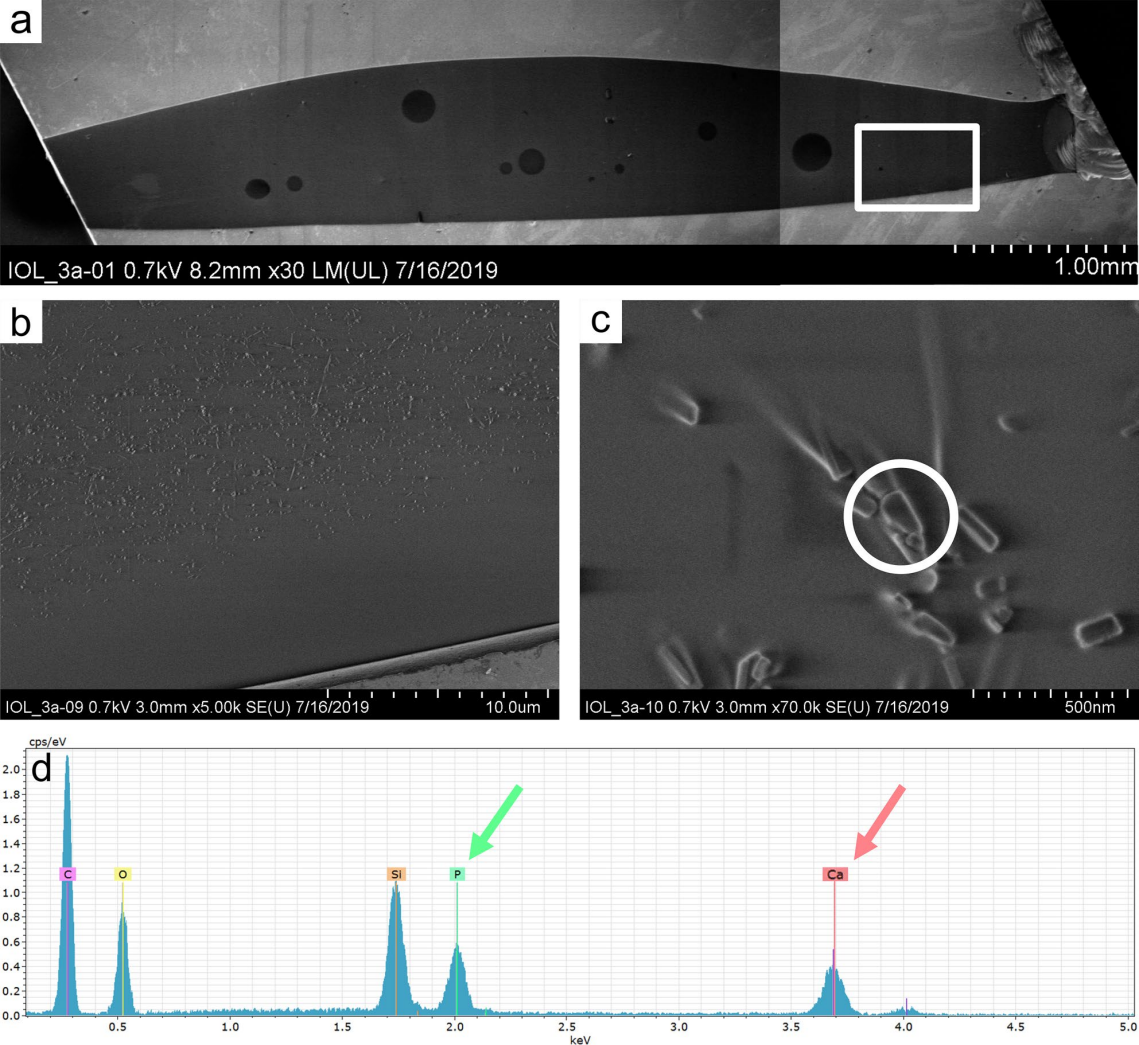
Chemical analysis of the crystals formed within the polymer of IOL 3a. Scanning electron microscopy investigation of a vertical section (**a**) overview, (**b**, **c**) higher magnification) with energy dispersive X-ray spectroscopy (**d**) confirms the crystals to consist of calcium (Ca) and phosphorus (P). The silicon peak (Si) originates from the silicon waver, carbon (C) and oxygen (O) peaks from the IOL polymer.

Light microscopy of Von Kossa stained vertical sections (Fig. 3c) and SEM with EDX investigation of vertical sections (Fig. 5) showed calcium phosphate crystals had also formed within the IOL polymer.

**Figure 6:**
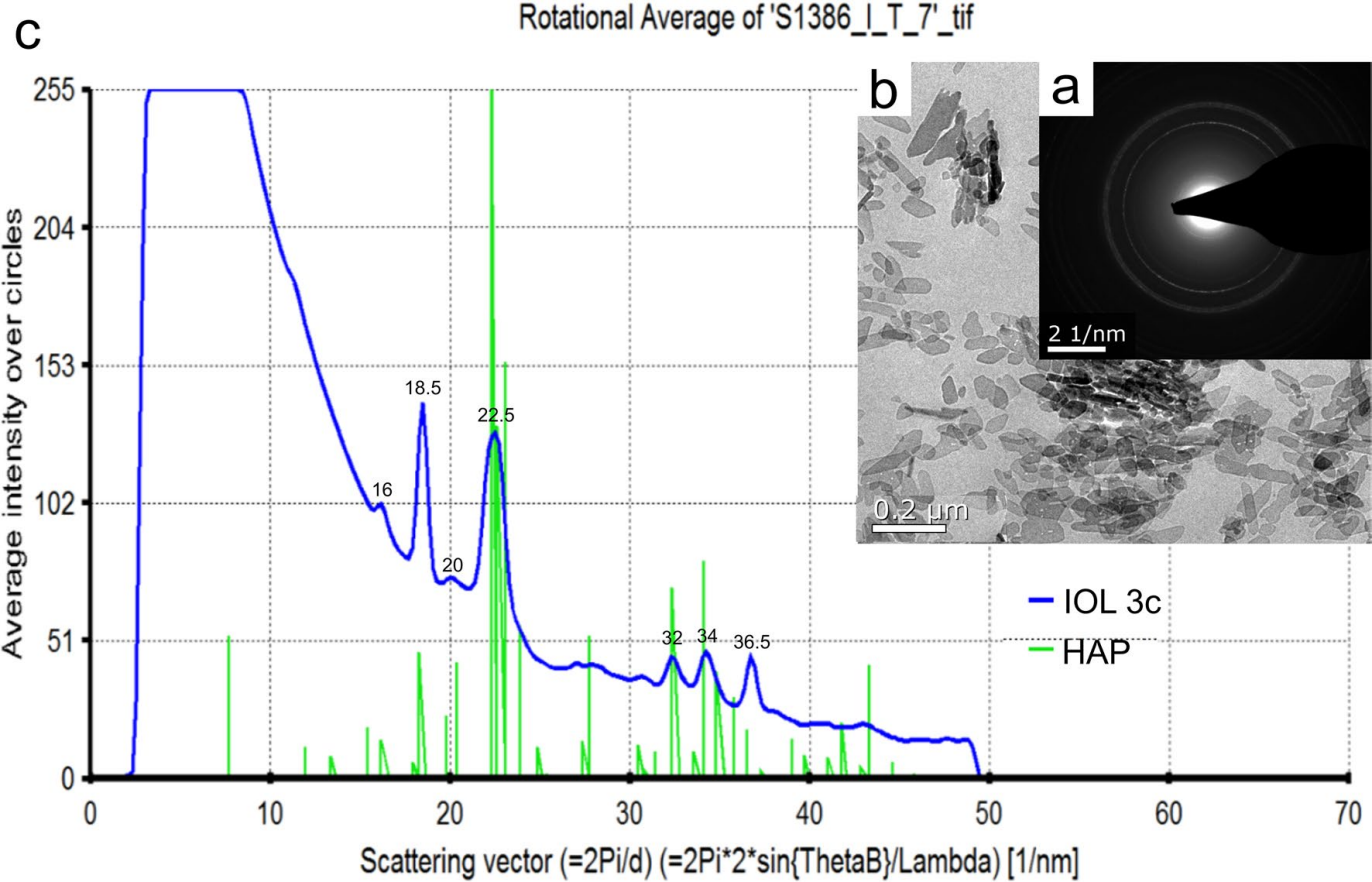
Electron crystallography of crystals within the polymer of IOL 3c. Transmission electron microscopy (**b**) provides the electron diffraction (ED) pattern of the crystals (**a**). Comparison (**c**) of the crystals’ ED pattern within IOL 3c to a reference ED of hydroxyapatite (HAP) shows a very high accordance: The ED of IOL 3c shows peaks at a scattering vector of 16, 18.5, 20, 22.5, 32, 34 and 36.5, characteristic for hydroxyapatite.

**Figure 7:**
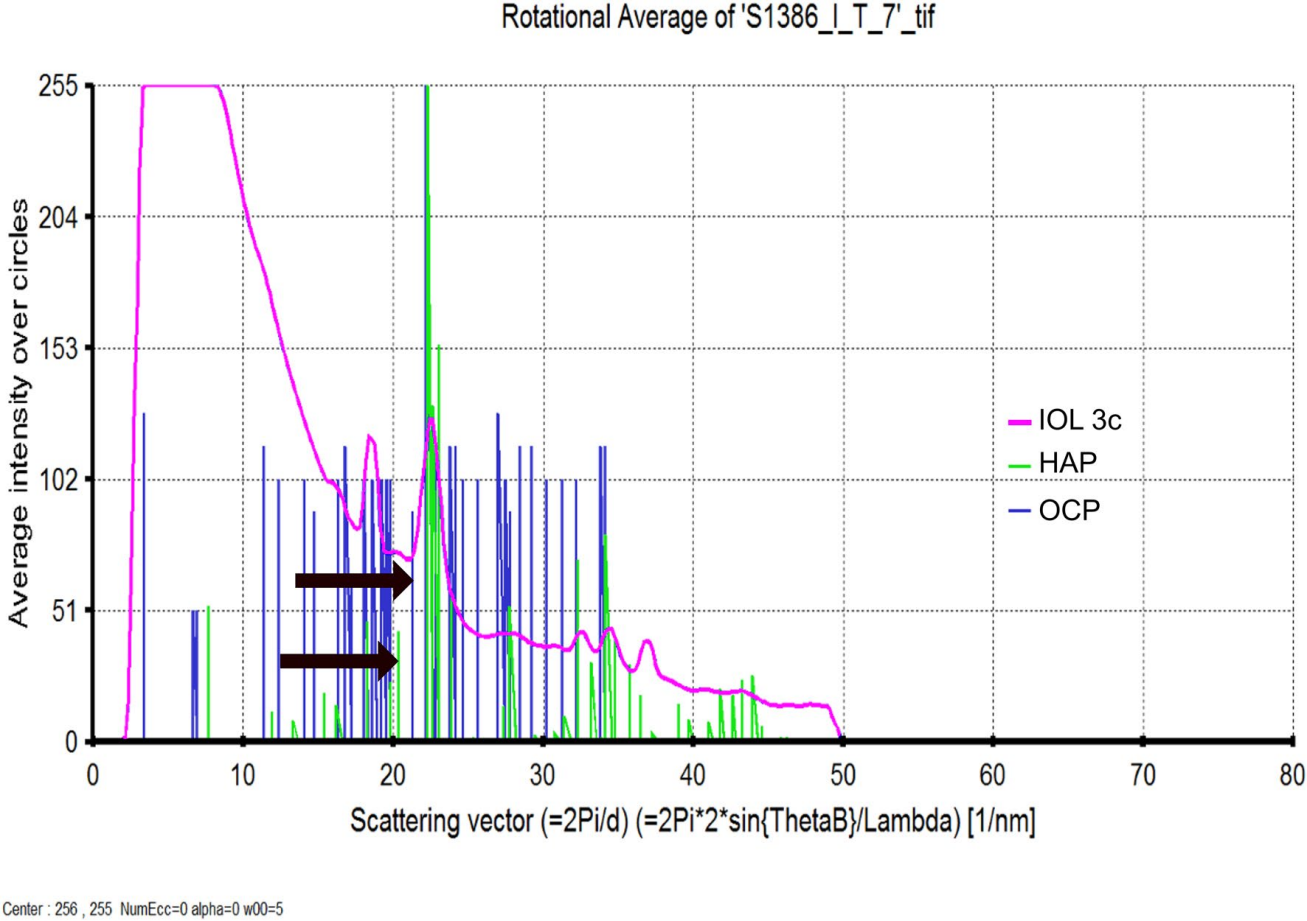
Distinction between crystals formed within the IOL polymer and hydroxyapatite (HAP) to octacalcium phosphate (OCP). Due to the OCP’s asymmetrical crystal structure, the reference electron diffraction (ED) pattern of OCP shows various diffraction reflections. These reflections are missing in the ED pattern of crystals in IOL 3c. Characteristic reflections to clearly distinguish OCP and HAP are marked (arrows).

Electron crystallography (Fig. 6) and comparison to both hydroxyapatite and the precursor octacalcium phosphate (Fig. 7) clearly showed the crystals within the polymer to be hydroxyapatite.

The hydrophobic IOLs showed surface calcium phosphate precipitation, but no crystal growth within the polymer. No noticeable difference in the occurrence of calcification could be found for the IOLs which manufacturers claim to have hydrophobic surface properties.

## Discussion

Calcification, the formation of hydroxyapatite crystals within the polymer of hydrophilic acrylic IOL, is a severe complication of cataract surgery^23,24^. When IOL calcification was first observed and documented^2^, little was known about the mechanisms causing it. In 2008, a classification based on clinical investigations was introduced by David Apple, distinguishing between primary and secondary calcification^3^. The former is related to the IOL polymer itself or arising from a problem in the manufacturing process. Secondary calcification, however, is associated with intraocular environmental changes, caused for example by surgical procedures (e.g., posterior lamellar keratoplasty, Pars-plana-Vitrectomy (PPV) or intracameral injection of recombinant tissue plasminogen activator) or linked to ocular and systemic disease (for example, diabetes mellitus)^6,20,25–29^.

More recent laboratory investigations have studied the underlying mechanisms of crystal formation in hydrophilic acrylic IOLs. Koutsoukos et al.^24^ showed two main reasons for IOL calcification. Firstly, the aqueous humor is a supersaturated solution of calcium and phosphate ions which favors precipitation. Over time, the ions diffuse into the hydrophilic IOL polymer. When the number of ions in solution in the polymer reaches saturation, the ions precipitate as a calcium phosphate salt. With further diffusion of calcium and phosphate ions into the polymer, the precipitated salts act as nuclei for crystallization^30–32^.

Secondly, the material used for the manufacture of hydrophilic acrylic IOLs plays a key role. The major component of this material is commonly Poly(2-hydroxyethyl methacrylate) (PHEMA). Carboxyl- and hydroxyl groups give this polymer polar properties, as they have a partial negative charge^24^. This negative charge leads to complexation of positively charged calcium ions and thereby favors diffusion, accumulation and precipitation with phosphate ions within the polymer^33^. Calcification might therefore be theoretically possible in every hydrophilic acrylic IOL containing PHEMA. To avoid this complication, surgeons could use only hydrophobic IOLs where calcification is rather unlikely^34^. The main component of hydrophobic IOLs is often Poly(methyl methacrylate) (PMMA) or Poly(ethyl acrylate) (PEA), which in comparison to PHEMA do not have polar hydroxyl groups and water content of hydrophobic IOLs is low (< 2%)^35^. Thus, diffusion and accumulation of calcium and phosphate ions within the hydrophobic polymer occur to a much lesser extent, if at all, so crystal formation is improbable. This is supported by the findings of a much lower hydroxyapatite growth rate on PMMA than on PHEMA^23^. However, hydrophobic IOLs can also develop material alterations (e.g., glistenings)^36^.

Furthermore, hydrophilic acrylic IOLs continue to be used because of their many advantages in terms of biocompatibility, ease of implantation, ease of alignment of the lens (especially important in toric IOL implantation where the lens is rotated within the capsule), the material’s compressibility during micro-incisional surgery and their low costs^37^.

The main material component of the hydrophilic IOLs tested in this study was PHEMA. We found that those IOLs described as having hydrophobic surface properties (Table 2) also exhibited calcification, the surface did not impede crystallization. This finding is in agreement with previous reports^15,16,24,38^ and it suggests that hydrophobic surfaces cannot prevent the diffusion of the ions into the polymer.

Diffusion of ions in solids is a slow process, and therefore calcification is typically a course over time rather than an immediate event^39^. Thus, a delay might be expected in the patient showing symptoms related to the lens opacification caused by calcification. However, there are case reports and epidemiological studies of conditions under which IOL calcification is reported to occur earlier^6,20,23,26^. Such risk factors accelerating calcification seem to be solutions high in phosphate concentration coming in contact with the IOL during the manufacturing process^15,16,40^, certain ophthalmic viscosurgical devices^41,42^, surgical procedures^4,20,26^ or physiological changes in the aqueous humor^43^. The physiological amount of phosphate in the aqueous humor is 2,19 mg/dl and typically lower than the serum concentration (3,27 mg/dl)^43^. Intraocular inflammation in patients with proliferative diabetic retinopathy (PDR), hypertension or post-surgery inflammation may alter the blood-aqueous barrier^5,25^. As a result, the diffusion of phosphate ions from the blood to the aqueous humor might be increased. This assumption is supported by the finding of significantly higher concentrations of phosphate ions in the aqueous humor of patients with PDR (3,03 mg/dl)^43^. The higher the concentration in the aqueous humor surrounding the IOL, the faster the ions will diffuse into the IOL and accelerate calcification. Another reason for elevated concentrations of phosphate in aqueous humor could be residual cataractous-lens material resulting from inadequate cortex cleaning^23^.

Therefore, we think one should rather talk about risk factors leading to premature calcification than strictly referring to primary or secondary calcification, as the underlying mechanism is most probably the same. The influence of risk factors might accelerate diffusion and therefore reduce the time needed until the solubility product is exceeded and crystallization starts.

IOL calcification has also been reported to occur after intraocular gas injection in PPV or posterior lamellar keratoplasty, where gas contact to the IOL might lead to dehydration and degeneration of the IOL’s surface^4,13,25,26^. It has been proposed that surface defects, coming in contact with the aqueous humor, can cause sedimentation^44^. Calcification preventive techniques with local saline solution irrigation of the anterior chamber to rinse of calcium ions out of the IOL polymer have been reported^45^. However, these techniques do not propose long-term solutions, since calcium ions are ubiquitously present in the aqueous humor and will diffuse back into the IOL polymer after irrigation.

IOL calcification appears to be a multifactorial phenomenon and in vitro models provide an approach to understanding the underlying mechanism. Existing methods to replicate IOL calcification^39^ are using a bioreactor simulating the anterior chamber, where experimental IOLs are placed in a solution equivalent to the aqueous humor. A syringe pump and a thermostat provide the physiological temperature and constant flow of the aqueous humor. Through slow diffusion of calcium and phosphate ions into the polymer, after five months, hydroxyapatite formed within the polymer. Other experimental models investigated the influence of viscoelastic materials and fatty acids on octacalcium phosphate surface nucleation of hydrophilic IOLs but did not induce hydroxyapatite growth within the polymer^42^. In vivo animal models to investigate calcification of different IOL materials also exist but have long trial run times of 10 months^46^.

We developed an in vitro model using electrophoresis to accelerate the diffusion and reproduced IOL calcification within 20 h. We varied the electrophoretic settings to define standard conditions (run 3 and 5) under which crystal formation and analysis were best possible. By standardizing the in vitro model, we hope to investigate the influence of risk factors affecting crystal formation. Moreover, our scope was to develop a fast-screening method, which simulates a worst-case scenario under harsh conditions to identify materials and IOL models at risk to calcify rather than replicating the exact in vivo conditions. Electrophoresis models have already been used before to create fast crystal growth in hydrophilic polymers^21,22,31^, but to the best of our knowledge, this approach has never been applied to IOLs. Light microscopy and SEM with EDX analysis confirmed the presence of calcium phosphate crystals on the IOL’s surface and within the polymer (Figs. 3, 4 and 5). A morphological comparison of in vitro calcified IOLs to explanted, in vivo calcified IOLs is not appropriate because the calcification pattern in in vivo explants is inhomogeneous^4,24^. To enable a comparison, the determination of the calcium phosphate crystal type would be the correct approach.

Different crystalline phases of calcium phosphate salts have been discussed to play a role in the process of calcification^39,47^. It has been shown that the thermodynamically most stable calcium phosphate hydroxyapatite is the main form to be found within the polymer of in vivo calcified IOLs^9,23^. However, octacalcium phosphate, a metastable precursor of hydroxyapatite, is discussed to build at an early state and eventually transform into hydroxyapatite with time.

Electron crystallography of the crystals formed within the polymer was performed. Comparison of these crystals’ electron diffraction pattern to hydroxyapatite (Fig. 6) and octacalcium phosphate (Fig. 7) identified these crystals to be hydroxyapatite.

Whereas in vivo calcification takes up to years^4^, this in vitro model creates an accelerated calcification process using harsh conditions. We thereby expect that we can use this electrophoresis setup as a fast test to identify hydrophilic IOLs that are at risk to develop calcification. Several implications are possible: varying and documenting time or concentration of the solution needed for calcification to occur for each IOL model, we could distinguish models prone to calcification. Furthermore, we could also investigate whether the influence of various risk factors will reduce time or concentration needed until calcification is measurable. Instead of measuring time or concentration, quantification of the crystals as a parameter of calcification would also be conceivable^44^. Further investigations and studies are required for this purpose.

In conclusion, we present a novel experimental model to replicate IOL calcification in vitro, in effort to contribute to a deeper understanding of this complication.

## Data Availability

The datasets generated during and/or analysed during the current study are available from the corresponding author on reasonable request. The schematic illustrations shown in Figs. 1 and 2 have been drawn by L.B. using Microsoft Power Point version 16.43. Reference electron diffraction pattern of HAP and OCP in Figs. 6 and 7 were obtained from PDF-2 2007; ICDD Newtown Square, PA, 2018.
